# Exposure of the heart and cardiac valves in women irradiated for breast cancer 1970–2009

**DOI:** 10.1016/j.ctro.2022.07.004

**Published:** 2022-07-16

**Authors:** Frances K. Duane, Naomi B. Boekel, Judy N. Jacobse, Zhe Wang, Berthe M.P. Aleman, Sarah C. Darby, Michael Schaapveld, Flora E. van Leeuwen, Margreet H.A. Baaijens, Samantha Warren, Carolyn W. Taylor

**Affiliations:** a St. Luke’s Radiation Oncology Network, St. Luke’s Hospital, Dublin, Ireland; b School of Medicine, Trinity College Dublin, Ireland; c Trinity St James’s Cancer Institute, St James’s Hospital, Dublin, Ireland; d Netherlands Cancer Institute, Epidemiology, Amsterdam, The Netherlands; e Nuffield Department of Population Health, University of Oxford, UK; f Netherlands Cancer Institute, Radiation Oncology, Amsterdam, The Netherlands; g Department of Radiotherapy, Erasmus MC Cancer Institute, University Medical Centre, Rotterdam, The Netherlands; h Northern Centre for Cancer Care, Freeman Hospital, Newcastle Upon Tyne, UK

**Keywords:** Radiation-related heart disease, Historical dosimetry, Radiation doses valves, Dose–response relationships

## Abstract

**Purpose:**

To describe cardiac exposure from breast cancer radiotherapy regimens used during 1970–2009 for the development of dose–response relationships and to consider the associated radiation-risks using existing dose–response relationships.

**Material and methods:**

Radiotherapy charts for 771 women in the Netherlands selected for case control studies of heart disease after breast cancer radiotherapy were used to reconstruct 44 regimens on a typical CT-dataset. Doses were estimated for the whole heart (WH), left ventricle (LV) and cardiac valves.

**Results:**

For breast/chest wall radiotherapy average WH doses decreased during 1970–2009. For internal mammary chain (IMC) radiotherapy WH doses were highest during the 1980s and 1990s when direct anterior fields were used and reduced in the 2000s when oblique fields were introduced. Average doses varied substantially for IMC regimens (WH 2–33 Gy, LV < 1–23 Gy). For cardiac valves, at least one valve received >30 Gy from most regimens.

**Conclusions:**

Radiation-risks of IHD from breast/chest wall regimens likely reduced during 1970–2009. Direct anterior IMC regimens likely increased the risks of IHD and VHD over this time period but the use of oblique IMC fields from 2003 may have lowered these risks. These data provide a unique opportunity to develop dose–response relationships.

## Highlights

• Cardiac exposure decreased substantially 1970–2009.
• Direct megavoltage IMC beams likely increase the risks of IHD and VHD.
• Cardiac dosimetry from past regimens is highly heterogeneous.
• Dosimetry from past decades is key for dose–response relationships for late effects.
• A wide variation in valve doses may enable a dose–response relationship for VHD.

## Introduction

Radiotherapy for breast cancer improves survival [1], [2] but may increase the risk of heart disease [3], [4], [5], [6], [7]. Most of the radiation-related risk is due to ischaemic heart disease (IHD) [3], [4], [5], [6], while for heart failure (HF) and valvular heart disease (VHD) increased risks have been reported in some populations of women [3], [5], [7], [8], [9] but not others [10], [11], [12], [13], [14]. Cardiac dose distributions in breast cancer radiotherapy vary for different regimens and in different populations [9], [15], [16], [17], [18], [19], [20] and differences in radiation-related risks of IHD, HF and VHD may be caused by differences in whole heart (WH), left ventricle (LV) and valve radiation doses.

Dose-response relationships suggest that there is little risk of radiation-related VHD from doses <30 Gy [21]. In contrast, the risk of IHD increases linearly by 6–7%/Gy [4], [6], with no evidence of a threshold below which, there is no increased risk. For HF a recent study showed that for women not receiving anthracyclines, radiotherapy was not associated with increased risk but, for women treated with anthracyclines, the risk increased according to radiation dose [7].

Radiation-related heart disease can take years to develop and historical data may hold important clues about radiation-related heart disease, clues that may not be provided by future studies of contemporary breast radiotherapy. Breast cancer regimens used to irradiate the internal mammary chain (IMC) in the Netherlands during 1970–2009 were associated with significantly increased risks of IHD, HF and VHD relative to non-IMC regimens [10]. Similar IMC regimens were used in other countries across Europe and the USA [15], [16], [18].This study aims to describe radiation doses to the WH, LV and cardiac valves from regimens used in the Netherlands 1970–2009 for the development of dose–response relationships. The associated radiation-related risks are considered.

## Methods and materials

Regimens were identified from the radiotherapy charts of women aged <71 years when diagnosed with Stage I-IIIA breast cancer or ductal carcinoma in situ selected for case-control studies of heart disease after breast cancer radiotherapy and included 296 cases and 475 controls [6], [7], [9]. Cases were women irradiated for breast cancer who had a cardiac event (myocardial infarction or heart failure). Information extracted included: field borders, surgery type, target, intended target dose, applied total dose, dose per fraction, beam energy and the use of shielding, wedges and bolus.

### Reconstruction and dose calculation

A “typical CT-scan” was selected by reconstructing commonly-used regimens on ten CT-scans randomly selected from the radiotherapy database of women irradiated in 2010. The CT-dataset that was typical for heart dose, and did not have unusual anatomy, was selected as the “typical CT-scan” [20], [22]. The treatment position was supine, with both arms above the head. Slice thickness was 3 mm and no intravenous contrast was used. The WH, LV, pulmonary valve, aortic valve, mitral valve, and tricuspid valve were contoured using atlases all by the same radiation oncologist (FD) [23], [24]. Electron or megavoltage photon regimens were reconstructed using 3-dimensional treatment planning (Varian Eclipse^TM^ TPS version 10.0.39 (Varian Medical Systems, Palo Alto, USA)). Field borders, gantry angles, and custom blocks were guided by these lines and by photographs of the fields and digitally reconstructed radiographs. The analytical anisotropic algorithm was used to calculate doses for photon plans, while a Monte Carlo method was used for electron plans, and a pencil beam algorithm for cobalt plans. Mixed energy beams were used if the relevant beam energies were not available. The 0.10 cc calculation volume grid was used to calculate doses for all except the cobalt regimens, where the minimum calculation volume grid was 0.25 cc. Tabular differential dose-volume histograms (DVHs) were exported for cardiac structures. Manual planning was used to estimate doses from orthovoltage fields [20].

### Cardiac doses for typical techniques and cardiac doses for individual patients

Typical radiotherapy techniques were those received by at least five women (including left-sided and right-sided breast cancer regimens). Typical regimen cardiac doses were estimated using the most frequently prescribed total dose for that regimen. The regimen doses contribute to Table 1, Table A.1, Table A.5-A.7.

**Table 1:** Mean radiation doses to cardiac structures from typical breast cancer regimens used at the Netherlands Cancer Institute or the Erasmus MC Cancer Institute in the Netherlands during 1970–2009.

Highlighted regimens are those used to irradiate the breast/chest wall and/or the SCF/axilla but not the IMC. All of the other regimens included the IMC.

*For further details on radiotherapy regimens see webtable 1.

**Fig. 1:**
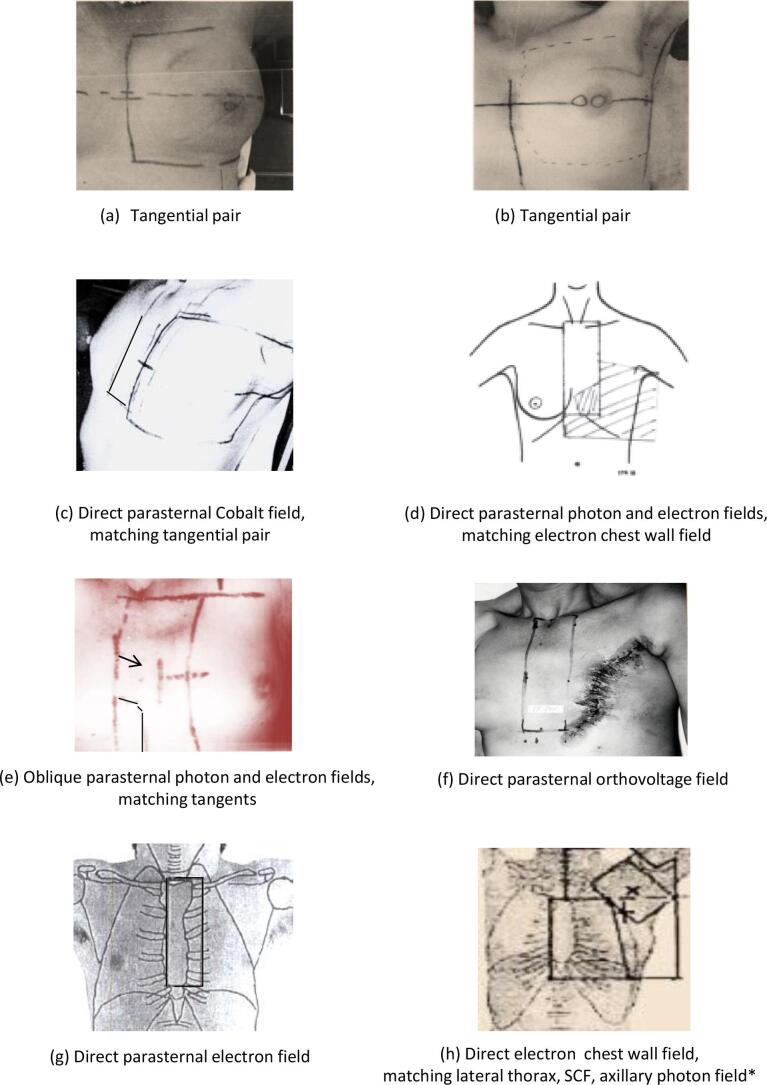
Radiotherapy fields used to treat women with breast cancer at the Netherlands Cancer Institute or the Erasmus MC Cancer Institute in the Netherlands between 1970 and 2009. Abbreviations: IMC: internal mammary chain, SCF: supraclavicular fossa *A posterior axillary boost field was used for some women to achieve a therapeutic dose to the axilla.

†Regimens (a)-(h) are illustrated in Fig. 1.

‡Mean cardiac doses estimated using manual planning are given to nearest Gy.

§Usual total dose (100%) to the target regions (see webtable 1 for dose ranges). For direct regimens this was the Dmax. For tangential regimens this was the dose delivered to the centre of the breast or chest wall apart from orthovoltage tangents where the total dose was the skin dose at the surface of the breast.

|| Cardiac doses from regimens used to irradiate left-breast cancer and right-breast cancer.

Abbreviations: IMC: internal mammary chain keV: kilovoltage, MV: megavoltage; MeV: mega electron-volts, SCF: supraclavicular fossa, contra: contralateral, ipsi: ipsilateral, Co^60^: cobalt 60.

Individual patient doses were also estimated for all women, including those who received atypical regimens, using the individual prescribed doses to different target regions. These individual patient doses were used to estimate changes in cardiac doses over time and relationships between LV and WH doses. The individual patient doses contribute to Fig. 4, Fig. 5, Table A.2- A.4, Table A.8.

**Fig. 2:**
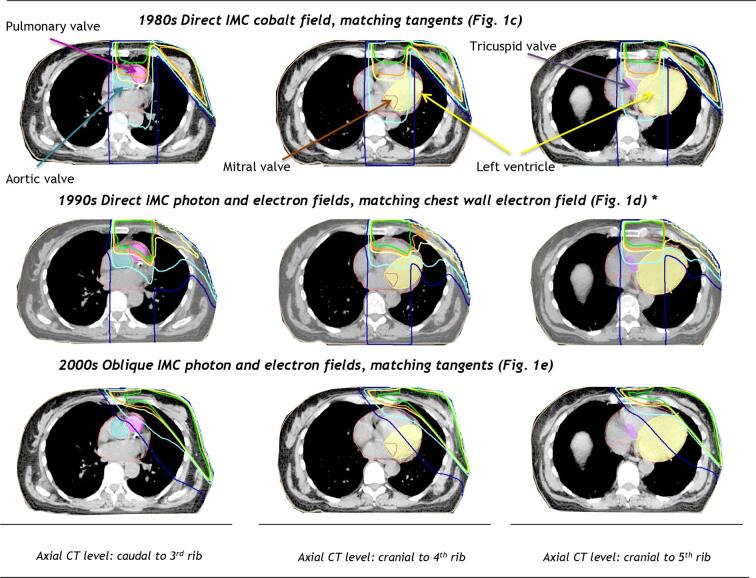
Spatial distribution of radiation dose to the heart from typical left-sided breast cancer radiotherapy regimens used in previous decades to target the breast or chest wall and internal mammary chain at the Netherlands Cancer Institute or the Erasmus MC Cancer Institute in the Netherlands. Isodoses (%): , , , ,  *Right-sided regimen is illustrated in Fig. 3. See Fig. 1. for illustrations of radiotherapy fields for regimens c-e. Abbreviations: IMC: internal mammary chain.

**Fig. 3:**
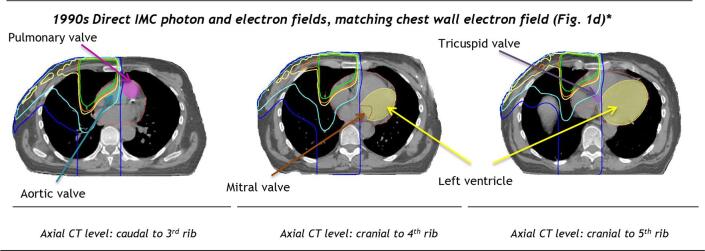
Spatial distribution of radiation dose to the heart from a right-sided breast cancer radiotherapy regimen. The treatment fields included mixed IMC photon and electron fields each giving 50% of the total dose respectively matched to an electron chest wall field. Isodoses (%): , , , , . See Fig. 1 for illustration of radiotherapy fields for regimen d. Abbreviations: IMC: internal mammary chain.

**Fig 4:**
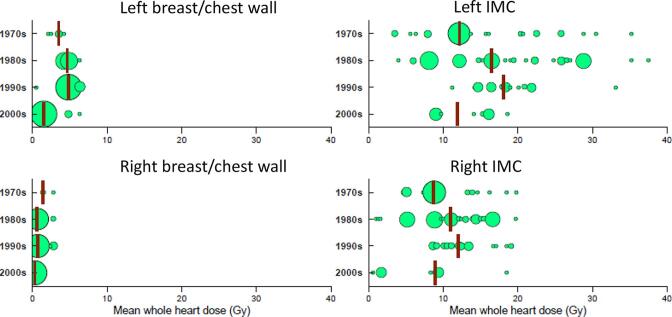
Whole heart radiation doses according to decade for 771 individual women who underwent radiotherapy for breast cancer at the Netherlands Cancer Institute or the Erasmus MC Cancer Institute in the Netherlands during 1970–2009. The median mean heart dose is shown for each decade by a vertical line. Each circle represents a group of women who received a similar heart dose, the area of the circle being proportional to the number of the women in the group. Abbreviation: IMC: internal mammary chain.

**Fig. 5:**
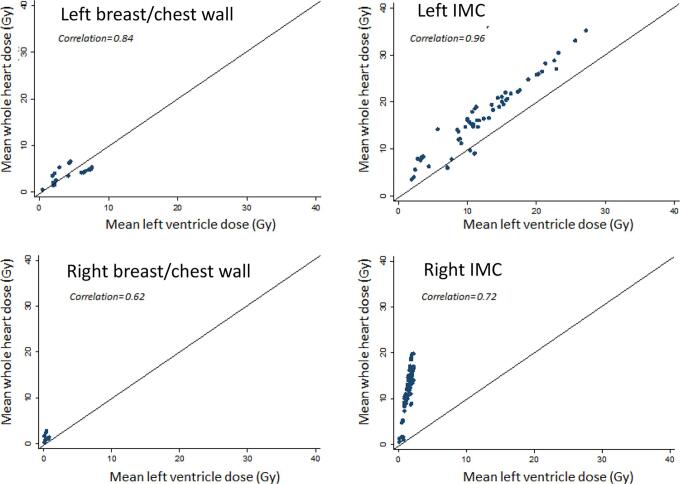
Correlations between whole heart and left ventricle mean radiation doses estimated for 771 individual women who underwent radiotherapy for breast cancer at the Netherlands Cancer Institute or the Erasmus MC Cancer Institute in the Netherlands during 1970–2009, by laterality and IMC irradiation.

DVHs were used to calculate mean organ doses for the WH, LV and cardiac valves. Dose-volume measures were calculated for the WH including: V_5Gy_ (percent volume receiving 5 Gy or more), V_10Gy,_ V_15Gy_ V_20Gy_ V_25Gy_ V_30Gy_ V_35Gy_ and V_40Gy._ For the WH and LV, mean doses in equivalent 2 Gy fractions (EQD2) were calculated for each individual dose-bin in each DVH using nd[(d + α/β)/(2 + α/β)], where n was the number of fractions, d was the mean dose to the cardiac structure per fraction (Gy), and α/β was 2 Gy [25], [26]. Then the individual dose-bin EQD2s were summed to estimate the total EQD2 for each structure.

## Results

There were 771 women selected for the study irradiated during 1970–2009. Twenty-two typical techniques were received by 754 women (44 regimens comprising 22 left-sided regimens and 22 right-sided regimens) (Table 1, Table A.1). Nine atypical techniques were identified received by 17 women.

Eight tangential regimens were used to irradiate the breast/chest wall (Table 1, Table A.1). For left megavoltage tangents used during 1970s-2000 (Fig. 1a) mean doses were: WH 4.8–5.3 Gy and LV 7.2–7.6 Gy. During this period opposing symmetrical tangential fields were used with the posterior borders divergent for some patients, aligned posteriorly for others and half-beam blocked for others. In general, the collimator was not angled to avoid the heart. For left-sided tangents used during 2000–2009 (Fig. 1b) mean doses were lower, WH 1.5 Gy and LV 2.1 Gy. During this period the posterior field edges were commonly aligned posteriorly or half beam blocked and the collimator was angled to avoid the heart. Left orthovoltage tangents used during 1970–1973 prescribed only 15.0 Gy skin dose, and all cardiac structures received ≤4 Gy. For right-sided tangential regimens, mean doses to all structures were <2 Gy. No cardiac valve received >30 Gy from tangential regimens.

Twenty-two direct anterior megavoltage regimens irradiated the IMC (Table 1, Table A.1, Fig. 1c-e, Fig. 2, Fig. 3). Mean cardiac doses were higher when the total dose to the IMC was given using a single megavoltage beam, compared with regimens with mixed megavoltage and electron fields. Mean doses for regimens using a single left megavoltage beam were: WH 20.4–33.1 Gy and LV 15.6–25.6 Gy. Valve doses varied from 25.1 to 51.8 Gy, with most valves receiving >30 Gy (Table 1, Fig. 2 top panel). For regimens with a single right anterior megavoltage beam, mean WH dose was 12.3–18.5 Gy. The aortic and tricuspid valves received >30 Gy from most regimens, whereas the pulmonary valve and the mitral valve were usually outside the fields and received lower doses.

For left-sided mixed direct megavoltage and electron IMC regimens (Fig. 2. middle panel) mean cardiac doses were: WH 14.7–20.7 Gy and LV 9.7–14.3 Gy. The cardiac valves received between 12.8 and 39.9 Gy, with the pulmonary valve receiving >30 Gy. For right-sided regimens (Fig. 3) mean WH dose was 8.5–12.0 Gy and tricuspid valve and aortic valve doses were 16.6–25.5 Gy. The pulmonary valve, mitral valve and LV were usually outside the fields and received ≤ 4.2 Gy. Oblique IMC fields were used after the year 2003 and resulted in lower cardiac doses (Fig. 2 bottom panel) (left-sided: mean WH dose 9.0 Gy, mean LV dose 11.0 Gy; right-sided: mean WH dose 1.7 Gy, mean LV dose 0.5 Gy). Mean doses to all valves were <30 Gy.

Six anterior orthovoltage or mixed orthovoltage/megavoltage IMC regimens were commonly used in the 1970s to 1990s to irradiate the IMC and supraclavicular fossa (Table 1, Table A.1, Fig. 1f). These fields were usually delivered alone, without breast or chest wall irradiation and resulted in WH doses of 9–22 Gy, LV doses of 2–16 Gy and valve doses of 3–38 Gy.

Eight anterior electron regimens were used to irradiate the chest wall and/or the regional lymph nodes (Table 1, Table A.1, Fig. 1g,h). The cardiac doses from these beams depended on beam energy. Cardiac doses were higher for the 12 MeV IMC fields (Fig. 1g) (left-sided: WH 7.9 Gy, LV 2.8 Gy) than for the 9 MeV chest wall fields (Fig. 1h) (left-sided: WH 4.2 Gy, LV 2.3 Gy). Of the cardiac valves the pulmonary valve received the highest doses (14.2–25.5 Gy) from left-regimens. For right-regimens the aortic valve received the highest doses (1.3–10.0 Gy). None of the valves received >30 Gy.

Individual patient doses for all 771 women were used to assess trends over time. Mean WH dose decreased over time, since fewer women received IMC radiation in the more recent decades (Table A.2). IMC radiotherapy decreased according to calendar year: 92% of the charts in the 1970s compared to 31% in the 2000s. Average WH dose in the 1970s and the 1980s was 8.9 Gy for both time periods. It reduced to 4.8 Gy in the 1990s and then to 1.5 Gy in the 2000s (Table A.2). Doses per decade were similar for women selected as cases or controls for the case-control studies^6-7^ (Table A.3-A.4).

Analyses that subdivided the women according to targets irradiated showed that mean WH dose reduced according to decade in breast/chest wall radiotherapy but not in IMC radiotherapy (Fig. 4, Tables A.2,A.3-A.4). For IMC radiotherapy, mean WH dose increased steadily during the 1970s, 1980s and 1990s. In the 1970s it was common to treat the IMC with a single parasternal field (which was often an electron field) to a total dose of 40–45 Gy (Table 1, Fig. 4). In the 1980s and 1990s direct IMC fields, either electron fields or megavoltage fields were often matched to breast/chest wall fields with prescribed dose 45–50 Gy (Table 1, Fig. 4), which contributed to increased exposure. During the 2000s, direct megavoltage fields continued to be used in some women, but others received oblique IMC fields, which were angled away from the heart. WH dose from 2000s IMC radiotherapy was similar to that from 1970s radiotherapy.

Correlations between LV and WH doses were seen in both left-sided (r = 0.84–0.96) and right-sided radiotherapy (r = 0.62–0.72) (Fig. 5). For left-sided non-IMC regimens, mean WH doses were similar to, or lower than, mean LV doses (WH 3.8 Gy (IQR 1.6–4.8), LV 5.2 Gy (IQR 2.2–7.2)) indicating that much of the cardiac dose was received by the LV. For left-sided IMC regimens mean WH doses were mostly higher than mean LV doses (WH 15.9 Gy (IQR 12.0–19.6), LV 11.6 (IQR 8.9–14.4 Gy) indicating that most of the cardiac dose was distributed to structures outside the LV. For right-sided radiotherapy LV doses were always lower than WH doses because the LV was usually several cm from the fields (Fig. 3).

## Discussion

Cardiac exposure from 44 regimens in 771 women irradiated in the Netherlands during 1970–2009 resulted in a wide spread of WH, LV and valve doses. Most breast/chest wall regimens delivered doses of <4 Gy to all structures compared to most IMC regimens which delivered doses of 5–30 Gy to the WH and LV, and >30 Gy to at least one cardiac valve. This may explain why women who received IMC regimens had significant excesses of IHD, HF and VHD relative to women who received non-IMC regimens in population data [10]. Dosimetry of past regimens form the backbone of dose response relationships developed in conjunction with long-term follow up data for assessment of risks for women today.

Our study has several strengths. The radiotherapy charts of 771 women including, on average, 17 charts per regimen were obtained and reconstructions had input from a radiation oncologist who had delivered them (BA). Heart doses estimated in this study for 2000s IMC radiotherapy are similar to published doses for similar regimens (Table A.7). There is a paucity of data in the literature relating to cardiac valve doses from breast cancer radiotherapy. A limitation is that individual anatomical information (e.g. CT-planning scans) was unavailable so the typical CT dataset method was used to estimate doses which are therefore subject to uncertainties [17], [18], [20], [27]. The main one is interpatient variability in anatomy. In a previous study WH dose varied between women by 7–9 Gy for left tangents, 3–5 Gy for left electron fields, ∼1 Gy for right tangents and 1–5 Gy for right electron fields [20]. Contouring variation may also lead to uncertainties in WH and LV doses of ∼1 Gy [23], [24]. Other sources of uncertainty include set-up error, inter- and intra-fraction motion, and dose-calculation algorithm error [28], [29], [30], [31], [32], [33]. Uncertainties are similar to other reconstruction methods [22].

In breast/chest wall radiotherapy, doses to all cardiac structures reduced between the 1970s and the 2000s. Breast/chest wall regimens used in the 1970s-1990s delivered 4–5 Gy to the WH. This dose may have increased the 30-year absolute risk of IHD for a typical patient by around 1–2 percentage points [4], [6]. Tangents used in the 2000s delivered <2 Gy WH dose, so the expected radiation-risks would be lower [4]. No cardiac valve received >30 Gy from any breast/chest wall regimen so these regimens are unlikely to have increased the risk of VHD [21].

In contrast, heart doses from IMC radiotherapy were higher and, because of the use of direct megavoltage fields, they did not reduce much during the 1970s to 2000s. A dose of around 12 Gy in left-IMC radiotherapy in the 2000s would be expected to nearly double a woman’s risk of ischaemic heart disease, which may increase the typical absolute 30-year risk by a few percentage points [4], [6]. In a systematic review of heart doses the corresponding values published worldwide during 2003–2013 were 8.4 Gy (range <1–29 Gy) for left-sided and 4.2 Gy (range 0.8–21.6) for right-sided IMC radiotherapy [34]. Hence the radiation-risks in different countries are likely to vary substantially. The proportional increase in the risk of heart disease for women irradiated 1970–2009 in the Netherlands will be similar to that in countries where similar regimens were used [14], [15], [18]. In other countries, regimens with lower heart doses are used [17], [28] and the radiation-related risks will be lower. Since the women in this study were irradiated, the increasing use of IMC fields angled away from the heart, the use of DIBH and the use of VMAT for selected cases has further reduced cardiac exposure, and doses are much lower for women irradiated in the Netherlands today [35].

The risk of radiation-related VHD increases steeply above 30 Gy [21]. Our findings suggest that most IMC regimens delivered >30 Gy to at least one of the cardiac valves. Some left IMC regimens delivered >30 Gy to all four valves whereas right IMC regimens usually only delivered >30 Gy to the tricuspid valve. These regimens are likely to have increased the risks of VHD in breast cancer survivors. This may be taken into account by physicians leading survivorship or cardio-oncology clinics.

In this study population the risks of several types of heart disease were raised [9], [10]. WH dose varied from <1 to 33 Gy while LV dose varied from ∼0.5 to 26 Gy. This has enabled IHD rates and HF rates to be compared across a wide spread of WH and LV doses and enabled dose–response relationships for radiation-induced IHD and HF to be estimated [6], [7]. Mean WH dose was a better predictor of heart disease than mean LV dose in both studies. At present, there are no dose–response relationships for the risk of VHD after breast cancer radiotherapy. The wide variation in valve doses in our study suggests comparison of VHD rates in these women may enable the development of a dose–response relationship. In a case-control study of patients with Hodgkin lymphoma the risk of radiation-related valve disease was increased among patients receiving >30 Gy to the valves. The relationship between valve dose and VHD may be different in patients with breast cancer because patients are older on average, have a higher likelihood of having co-existing risk factors and receive different regimens, with differing dose fractionation schedules and cardiac dose distributions. Furthermore, the substantial variation in exposure of all four valves from various regimens may allow investigation into the varying sensitivity of individual valves to radiation using this population. In the general population most heart valve problems involve the left-sided aortic and mitral valves [36]. These are also the most commonly affected valves in breast cancer survivors [8] and other patient groups who received mediastinal radiotherapy [37], [38], [39]. It would be of interest to determine if the right-sided pulmonary valve or tricuspid valves are also damaged by radiation, or whether it is only the left-sided aortic and mitral valves that need to be avoided during radiotherapy treatment planning. Since the implementation of cardiac-sparing techniques, cardiac exposure is much reduced nowadays for most women. Nevertheless, information on sensitivity of the cardiac valves would inform radiotherapy planning for the few women with high cardiac exposure despite advanced techniques, as well as for other patient groups receiving thoracic radiation.

In conclusion, cardiac dosimetry from past regimens is highly heterogeneous, providing a unique opportunity for the development of dose–response relationships for assessment of risks for women today. Patients who received IMC regimens which included direct megavoltage beams are likely to have increased the risks of IHD and VHD. In contrast, breast/chest wall regimens used in the 2000s are unlikely to have increased the risks of valve disease and the absolute 30-year radiation-risks of incident IHD are likely to be <1 %.

## Declaration of Competing Interest

The authors declare the following financial interests/personal relationships which may be considered as potential competing interests: Dr. Warren reports non-financial support from Raystation UK Users Meeting May 2019, outside the submitted work.
