# Supplementary material for: Exposure of the heart and cardiac valves in women irradiated for breast cancer 1970–2009
Source: Clin Transl Radiat Oncol. 2022 Jul 16;36:132–9. doi: 10.1016/j.ctro.2022.07.004 (PMC9399376; doi:10.1016/j.ctro.2022.07.004)
Supplement: Supplementary data 1 [file mmc1.pdf]

Table A.1. Typical radiotherapy regimens and dose-fractionation schedules for 754 women with breast cancer at the Netherlands Cancer Institute or the Erasmus MC Cancer Institute in the Netherlands during 1970-2009.

Years of radiotherapy	Field arrangement*	*No. of women*	*Beam energy*	Target(s)†	*Dose (Gy)§*	*Dose/ fraction*	Field borders	*Comments*
Tangential fields (n=290)								
1970-1973	Tangents	5	250 keV	Chest wall	15.0	3.0	Superior - clavicular head<br>Inferior - 1cm below inframammary fold<br>Medial - midline<br>Lateral - mid-axillary line	Posterior border typically divergent<br>Opposing symmetrical fields
1975-1989	Tangents	9	Co^{60}	Breast	50.0	2.0	Superior - clavicular head<br>Inferior - 1cm below inframammary fold<br>Medial - midline<br>Lateral - mid-axillary line	Posterior border typically divergent<br>Opposing symmetrical fields<br>Collimator not angled<br>Fixed SSD 80cm
1981-2001	Tangents (Fig. 1a)	175	4-8 MV	Breast	44.0-52.0	2.0	Superior - clavicular head<br>Inferior - 1cm below inframammary fold<br>Medial - midline<br>Lateral - mid-axillary line	Posterior field edges divergent/aligned/half-beam blocked<br>Opposing symmetrical fields<br>Collimator not angled<br>Isocentric technique
2000-2009	Tangents (Fig. 1b)	101	6-18 MV	Breast/chest wall	46.2-51.5	1.7-2.5	Superior - clavicular head<br>Inferior - 1cm below inframammary fold<br>Medial - midline<br>Lateral - mid-axillary line	Posterior field edges aligned/half-beam blocked<br>Collimator angled to avoid heart<br>Many women received field-in-field radiotherapy
Megavoltage IMC fields (n=200)								
Megavoltage								
1971-1984	Direct IMC	8	Co^{60}	IMC	40.0-53.0	2.1-3.3	Superior - sternal notch<br>Inferior - xiphoid tip<br>Medial - 1cm from midline contra<br>Lateral - 5cm from midline ipsi	Gantry angle 0°<br>Fixed SSD 80cm
1973-1985	Direct IMC, matching chest wall	15	Co^{60}	IMC‡	39.6-40.0	2.5-3.3	Superior - sternal notch<br>Inferior - xiphoid tip<br>Medial - 1cm from midline contra<br>Lateral - 5cm from midline ipsi	Gantry angle 0°<br>Fixed SSD 80cm
			9 MeV	Chest wall	36.8-37.6	2.3-2.4	Superior - middle of 2nd costal cartilage<br>Inferior - 1cm below inframammary fold<br>Medial - matched to lateral border of IMC field<br>Lateral - mid-axillary line	Gantry angle 0°
1977-1989	Direct IMC, matching tangents (Fig. 1c)	45	Co^{60}	IMC‡	43.0-53.8	1.9-2.9	Superior - sternal notch<br>Inferior - xiphoid tip<br>Medial - 1cm from midline contra<br>Lateral - 5cm from midline ipsi	Gantry angle 0°<br>Fixed SSD 80cm
			6 MV	Breast	40.0-50.0	1.8-2.0	Superior - clavicular head<br>Inferior - 1cm below inframammary fold<br>Medial - matched to lateral border of IMC field<br>Lateral - mid-axillary line	Medial beam overlapped the IMC field by 5mm on skin

Years of radiotherapy	Field arrangement*	*No. of women*	*Beam energy*	Target(s)†	*Dose (Gy)§*	*Dose/ fraction*	Field borders	*Comments*
1975-1993	Direct IMC, direct SCF/axilla/lateral thorax	21	4-6 MV	IMC	40.1-56.3	2.2-3.0	Superior - suprasternal notch<br>Inferior - xiphoid tip<br>Medial - 1cm from midline contra<br>Lateral - 5cm from midline ipsi	Six women had a direct IMC field only
			8 MV	SCF/axilla/lateral thorax	44.5-56.3	2.2-2.5	Superior - inferior border of C6 vertebra<br>Infero-lateral - where rib 7 touches the lung contour<br>Infero-medial - rib 2<br>Medial - 1cm from midline contra<br>Lateral - 3cm lateral to humeral head	Medial beam overlapped the IMC field by 5mm on skin
1979-2006	Direct IMC, matching tangents	12	4-8 MV	IMC‡	16.0-52.0	2.0-2.5	Superior - suprasternal notch<br>Inferior - xiphoid process<br>Medial - 1cm from midline contra<br>Lateral - 5cm from midline ipsi	Gantry angle 0°
			4-25 MV	Breast	50.0-52.0	2.0	Superior - middle of 2nd costal cartilage<br>Inferior - 1cm below inframmary fold<br>Medial - matched to IMC field<br>Lateral - mid-axillary line	Medial beam overlapped the IMC field by 5mm on skin
Mixed megavoltage/electron								
1979-1987	Direct IMC, matching tangents	7	Co^{60}	IMC‡	15.4-36.0	1.5-2.0	Superior - sternal notch<br>Inferior - xiphoid tip<br>Medial - 1cm from midline contra<br>Lateral - 5cm from midline ipsi	Gantry angle 0°<br>Fixed SSD 80cm
			12 MeV	IMC‡	9.7-30.0	1.9-2.7	Superior - sternal notch<br>Inferior - xiphoid tip<br>Medial - 1cm from midline contra<br>Lateral - 5cm from midline ipsi	Gantry angle 0°
			6 MV	Breast	45.0-50.0	1.8-2.0	Superior - middle of 2nd costal cartilage<br>Inferior - 1cm below inframammary fold<br>Medial - matched to lateral border of IMC field<br>Lateral - mid-axillary line	Medial beam overlapped the IMC field by 5mm on skin
1981-1999	Direct IMC, matching tangents	15	6-8 MV	IMC‡	22.5-40.0	2.0-2.5	Superior - suprasternal notch<br>Inferior - xiphoid process<br>Medial - 1cm from midline contra<br>Lateral - 5cm from midline ipsi	Gantry angle 0°
			10-12 MeV	IMC‡	10.0-26.0	2.0-2.5	Superior - suprasternal notch<br>Inferior - xiphoid process<br>Medial - 1cm from midline contra<br>Lateral - 5cm from midline ipsi	Gantry angle 0°
			6-8 MV	Breast	47.5-50.0	2.0-2.5	Superior - middle of 2nd costal cartilage<br>Inferior - 1cm below inframammary fold<br>Medial - matched to IMC field<br>Lateral - mid-axillary line	Lateral tangent entered through the mid-axillary line to form the pair<br>Medial beam overlapped the IMC field by 5mm on skin

Years of radiotherapy	Field arrangement*	*No. of women*	*Beam energy*	Target(s)†	*Dose (Gy)§*	*Dose/ fraction*	Field borders	*Comments*
1991-2000	Direct IMC, matching chest wall (Fig. 1d)	22	4-8 MV	IMC‡	22.5-26.7	2.0-2.5	Superior - suprasternal notch<br>Inferior - xiphoid process<br>Medial - 1cm from midline contra<br>Lateral - 5cm from midline ipsi	Diagonal block below 4th rib<br>Gantry angle 0°
			10-12 MeV	IMC‡	22.5-26.0	2.0-2.5	Superior - suprasternal notch<br>Inferior - xiphoid process<br>Medial - 1cm from midline contra<br>Lateral - 5cm from midline ipsi	Diagonal block below 4th rib<br>Gantry angle 0°
			6-10 MeV	Chest wall	40.0-50.0	2.0-2.5	Superior - middle of 2nd costal cartilage<br>Inferior - 1cm below inframammary fold<br>Medial -superior - matched to lateral border of IMC field<br>Medial -inferior - midline sternum<br>Lateral - mid-axillary line	Gantry angle 0°
1993-2006	Direct IMC	15	6-8 MV	IMC	18.0-19.3	2.0-2.8	Superior - suprasternal notch<br>Inferior - xiphoid process<br>Medial - 1cm from midline contra<br>Lateral - 5cm from midline ipsi	Gantry angle 0°
			10-12 MeV	IMC	21.4-32.0	2.0-2.7	Superior - suprasternal notch<br>Inferior - xiphoid process<br>Medial - 1cm from midline contra<br>Lateral - 5cm from midline ipsi	Gantry angle 0°
2000-2005	Direct IMC, matching tangents	19	6-10 MV	IMC‡	15.8-18.0	2.0-2.3	Superior - cricoid cartilage<br>Inferior - xiphoid tip<br>Medial - 1cm from midline contra<br>Lateral - 5cm from midline ipsi	CT planned to deliver 85% of total dose to lymph nodes<br>Inferior border extended caudally to include the medial aspect of the breast
			8-17 MeV	IMC‡	32.0-33.8	2.1-2.3	Superior - cricoid cartilage<br>Inferior - xiphoid tip<br>Medial - 1cm from midline contra<br>Lateral - 5cm from midline ipsi	CT planned to deliver 85% of total dose to lymph nodes<br>Inferior border extended caudally to include the medial aspect of the breast
			6-10 MV	Breast/chest wall	49.6-50.0	2.0-2.2	Superior - middle of 2nd costal cartilage<br>Inferior - 1cm below inframammary fold<br>Medial - 4.5 cm from midline ipsi matched to IMC field<br>Lateral - mid-axillary line	Opposing symmetrical fields<br>Posterior beam edges aligned<br>Medial beam overlapped the IMC field by 5mm on skin

Years of radiotherapy	Field arrangement*	*No. of women*	*Beam energy*	Target(s)†	*Dose (Gy)§*	*Dose/ fraction*	Field borders	*Comments*
2003-2010	Oblique IMC, matching tangents (Fig. 1e)	21	6-10 MV	IMC‡	16.6-18.1	1.7-2.0	Superior - clavicular head<br>Inferior - xiphoid tip<br>Medial - 2cm from midline contra<br>Lateral - 4cm from midline ipsi	CT planned to deliver 85% of total dose to lymph nodes<br>Inferior border extended caudally to include the medial aspect of the breast
			10-20 MeV	IMC‡	32.0-34.9	1.7-2.1	Superior - clavicular head<br>Inferior - xiphoid tip<br>Medial - 2cm from midline contra<br>Lateral - 4cm from midline ipsi	CT planned to deliver 85% of total dose to lymph nodes<br>Inferior border extended caudally to include the medial aspect of the breast
			6-10 MV	Breast/chest wall	50.0-51.5	1.7-2.0	Superior - middle of 2nd costal cartilage<br>Inferior - 1cm below inframammary fold<br>Medial - 3.5 cm from midline ipsi matched to IMC field<br>Lateral - mid-axillary line	Opposing symmetrical fields<br>Posterior beam edges aligned<br>Medial beam overlapped the IMC field by 5mm on skin<br>Many women had field-in-field
**Orthovoltage or mixed orthovoltage/megavoltage IMC fields (n=172)**								
1971-1985	Direct IMC (Fig. 1f)	116	250 keV¶	IMC	36.2-48.8	2.2-2.7	Superior -sternal notch<br>Inferior - xiphoid tip<br>Medial - 1cm from midline contra<br>Lateral - 5cm from midline ipsi	Gantry angle 0°
1981-1989	Direct IMC	33	250 keV¶	IMC	17.2-21.5	1.8-2.5	Superior - sternal notch<br>Inferior - xiphoid tip<br>Medial - 1cm from midline contra<br>Lateral - 5cm from midline ipsi	Gantry angle 0°
			Co^{60}	IMC	19.1-31.9	2.1-2.9	Superior - sternal notch<br>Inferior - xiphoid tip<br>Medial - 1cm from midline contra<br>Lateral - 5cm from midline ipsi	Gantry angle 0°<br>Fixed SSD 80cm
1989-1992	Direct IMC	23	250 keV¶	IMC	16.8-23.5	1.8-2.5	Superior - sternal notch<br>Inferior - xiphoid tip<br>Medial - 1cm from midline contra<br>Lateral - 5cm from midline ipsi	Gantry angle 0°
			4-8 MV	IMC	21.9-24.0	2.0-2.7	Superior -sternal notch<br>Inferior - xiphoid tip<br>Medial - 1cm from midline contra<br>Lateral - 5cm from midline ipsi	Gantry angle 0°
**Electron chest wall or IMC fields (n=92)**								
1975-1985	Direct IMC (Fig. 1g)	36	10-16 MeV	IMC	45.0	3.0	Superior - sternal notch<br>Inferior - xiphoid process<br>Medial - 1cm from midline contra<br>Lateral - 5cm from midline ipsi	Gantry angle 0°<br>The direct MeV IMC field covered intercostal spaces 1-4

Years of radiotherapy	Field arrangement*	*No. of women*	*Beam energy*	Target(s)†	*Dose (Gy)§*	*Dose/ fraction*	Field borders||	*Comments*
1978-1985	Direct IMC, matching tangents	22	10-13 MeV	IMC	30.0-45.0	2.0-3.0	Superior - sternal notch<br>Inferior - xiphoid tip<br>Medial - 1cm from midline contra<br>Lateral - 5cm from midline ipsi	Gantry angle 0°<br>The direct MeV IMC field covered intercostal spaces 1-4
			6 MV	Breast	33.4-50.0	1.8-2.2	Superior - middle of 2nd costal cartilage<br>Inferior - 1cm below inframammary fold<br>Medial - matched to lateral border of IMC field<br>Lateral - mid-axillary line	Medial beam overlapped the IMC field by 5mm on skin
1970-1998	Direct chest wall	14	10-13 MeV	Chest wall||	40.1-60.0	2.0-3.0	Superior- matched to SCF/axilla field<br>Inferior - xiphoid process<br>Medial - 1cm from midline contra<br>Lateral - 1cm medial to lateral lung contour	Gantry angle 0°
1972-2003	Direct chest wall, direct SCF/axilla/lateral thorax (Fig.	20	8-13 MeV	Chest wall||	40.0-50.0	2.0-3.0	Superior - matched to SCF/axilla field<br>Inferior - xiphoid process<br>Medial - 1cm from midline contra<br>Lateral - 1cm medial to lateral lung contour	Gantry angle 0°
			8 MV	SCF/axilla/lateral thorax	43.0-53.0	2.0-2.7	Superior - inferior border of C6 vertebra<br>Infero-lateral - where rib 7 touches the lung contour<br>Infero-medial - rib 2<br>Medial - 1cm from midline contra<br>Lateral - 3cm lateral to humeral head	Gantry angle 0°<br>Large anterior chest wall block (lateral border 1cm medial to lateral lung contour)

*Radiotherapy regimens are ordered as in Table 1. Regimens a-h are illustrated in Fig. 1.
†Separate fields to the SCF and/or axilla were not reconstructed as they would have delivered <0.5 Gy mean heart dose. Separate boost fields were not reconstructed as information was lacking on the location of the surgical bed.
‡§For IMC fields matched to breast or chest wall fields the inferior border reached the 5th intercostal space including the xiphoid process in many cases. This was to allow coverage of the breast or chest wall tissue medially. The typical field size was 18-25 cm.
§Usual total dose (100%) to the target regions. For tangents the total dose was delivered to the centre of the breast or chest wall apart from orthovoltage tangents where the total dose was the skin dose at the surface of the breast. For direct regimens this was the Dmax.
¶Some orthovoltage IMC fields were matched to an electron chest wall field or a tangential pair to the breast. Cardiac doses for these field combinations were not calculated as it was not possible to create a radiotherapy plan using both manual planning and CT-planning.
||For many women who were treated with direct electron chest wall fields the internal mammary chain also received a therapeutic dose.
Abbreviations: IMC: internal mammary chain, keV: kilovoltage, MV: megavoltage; MeV: mega electron-volts, SCF: supraclavicular fossa, contra: contralateral, SSD: source-surface distance, AP: antero-postero, ipsi: ipsilateral

Table A.2. Whole heart and left ventricle radiation doses according to decade estimated for 771 individual women selected as cases and controls for case-control studies of heart disease after treatment for breast cancer who underwent radiotherapy at the Netherlands Cancer Institute or the Erasmus MC Centre in the Netherlands during 1970-2009.*

	Treatment period											
	1970s			1980s			1990s			2000s		
Regions	No.	Median mean dose (IQR)		No.	Median mean dose (IQR)		No.	Median mean dose (IQR)		No.	Median mean dose (IQR)	
irradiated	women	Whole heart	Left ventricle	women	Whole heart	Left ventricle	women	Whole heart	Left ventricle	women	Whole heart	Left ventricle
Left No IMC	**7**	**3.5 (3.0-3.5)**	**4.2 (2.2-4.2)**	**48**	**4.7 (4.3-4.8)**	**6.6 (6.5-7.2)**	**70**	**4.8 (4.8-4.8)**	**7.2 (6.7-7.2)**	**59**	**1.5 (1.5-1.6)**	**2.1 (2.1-2.2)**
Breast	1	4.2 (4.2-4.2)	6.1 (6.1-6.1)	43	4.8 (4.3-4.8)	7.2 (6.5-7.2)	60	4.8 (4.8-4.8)	7.2 (7.2-7.2)	55	1.5 (1.5-1.6)	2.1 (2.1-2.2)
Chest wall	6	3.5 (2.8-3.5)	3.3 (2.1-4.2)	5	4.8 (4.3-4.8)	6.7 (6.5-7.2)	10	6.3 (6.3-6.3)	4.3 (4.3-4.4)	4	1.5 (1.5-2.7)	2.1 (2.1-2.7)
Left IMC	**63**	**12.2 (12.0-12.2)**	**8.9 (8.7-8.9)**	**107**	**16.5 (8.3-25.8)**	**12.4 (4.5-20.0)**	**36**	**18.1 (16.0-20.3)**	**11.1 (9.9-14.4)**	**26**	**11.9 (9.0-16.1)**	**11.0 (11.0-11.4)**
IMC (No chest wall/breast)	44	12.2 (12.0-12.2)	8.9 (8.7-8.9)	56	15.9 (12.2-16.5)	12.0 (8.9-12.4)	13	14.7 (14.7-18.3)	9.7 (9.7-13.7)	1	16.1 (16.1-16.1)	10.0 (10.0-10.0)
IMC, breast	5	13.7 (12.2-15.7)	8.9 (8.7-10.3)	44	26.6 (8.3-28.8)	20.8 (3.4-22.6)	12	21.8 (19.8-21.9)	15.5 (14.1-16.3)	10	9.1 (9.0-13.9)	11.0 (10.7-11.1)
IMC, chest wall	14	12.2 (7.7-12.2)	8.9 (5.5-8.9)	7	22.3 (18.5-22.3)	17.4 (14.1-17.4)	11	16.4 (16.4-18.3)	10.0 (10.0-10.9)	15	16.1 (9.0-16.1)	11.1 (11.0-11.4)
Right No IMC	**3**	**1.4 (1.4-2.1)**	**0.8 (0.6-0.8)**	**38**	**0.6 (0.6-0.7)**	**0.2 (0.2-0.2)**	**49**	**0.7 (0.7-0.7)**	**0.2 (0.2-0.2)**	**47**	**0.3 (0.3-0.3)**	**0.1 (0.1-0.1)**
Breast	0	-	-	34	0.6 (0.6-0.7)	0.2 (0.2-0.2)	43	0.7 (0.7-0.7)	0.2 (0.2-0.2)	44	0.3 (0.3-0.3)	0.1 (0.1-0.1)
Chest wall	3	1.4 (1.4-2.1)	0.8 (0.6-0.8)	4	2.2 (1.6-2.8)	0.4 (0.3-0.4)	6	2.8 (2.8-2.8)	0.4 (0.4-0.4)	3	0.3 (0.3-0.3)	0.1 (0.1-0.1)
Right IMC	**59**	**8.7 (8.7-8.9)**	**1.8 (1.8-1.9)**	**100**	**11.0 (8.9-14.7)**	**1.8 (1.4-1.9)**	**38**	**12.0 (10.1-13.4)**	**1.3 (0.9-1.7)**	**21**	**8.9 (1.7-9.4)**	**1.0 (0.5-1.1)**
IMC (No chest wall/breast)	45	8.7 (8.7-8.9	1.8 (1.8-1.9)	55	8.9 (6.9-11.0)	1.8 (1.0-1.9)	9	8.7 (8.7-11.1)	0.9 (0.9-1.5)	2	8.9 (8.9-9.1)	1.0 (1.0-1.0)
IMC, breast	5	7.3 (4.7-13.2)	0.9 (0.5-1.7)	37	16.2 (11.0-16.6)	2.1 (1.4-2.2)	16	13.4 (12.4-14.2)	1.8 (1.3-2.0)	9	8.9 (1.7-9.4)	1.1 (0.5-1.1)
IMC, chest wall	9	8.9 (8.9-8.9)	1.9 (1.8-1.9)	8	12.3 (11.6-13.0)	1.6 (1.5-1.7)	13	10.5 (10.1-12.3)	0.9 (0.9-1.7)	10	1.7 (1.7-9.4)	0.5 (0.5-1.1)
All women	**132**	**8.9 (8.7-12.2)**	**2.1(1.8-8.9)**	**293**	**8.9 (4.8-16.5)**	**2.8 (1.6-8.9)**	**193**	**4.8 (2.8-12.4)**	**4.5 (0.4-7.2)**	**153**	**1.5 (0.3-6.3)**	**2.1 (0.1-2.2)**

*Radiotherapy charts from all 771 women included in two case-control studies of heart disease after breast cancer treatment - cases and controls. Doses per decade were similar when just the 475 controls were analysed (Webtable 3).

Abbreviations: IMC: internal mammary chain, IQR: inter-quartile range

Table A.3. Whole heart and left ventricle radiation doses according to decade estimated for 475 individual women selected as controls for case-control studies of heart disease after treatment for breast cancer who underwent radiotherapy at the Netherlands Cancer Institute or the Erasmus MC Centre in the Netherlands during 1970-2009.*

	Treatment period											
	1970s			1980s			1990s			2000s		
Regions	No.	Median mean dose (IQR)		No.	Median mean dose (IQR)		No.	Median mean dose (IQR)		No.	Median mean dose (IQR)	
irradiated	women	Whole heart	Left ventricle	women	Whole heart	Left ventricle	women	Whole heart	Left ventricle	women	Whole heart	Left ventricle
Left No IMC	3	3.5 (2.8-3.9)	2.0 (2.0-4.1)	33	4.3 (4.3-4.8)	6.5 (6.5-7.2)	54	4.8 (4.8-4.8)	7.2 (7.2-7.2)	46	1.5 (1.5-1.5)	2.1 (2.1-2.1)
Left IMC	39	12.2 (12.0-12.2)	8.9 (8.7-8.9)	65	16.5 (8.3-25.8)	12.4 (7.1-20.0)	21	16.4 (14.7-18.3)	10.0 (9.7-13.7)	18	9.1 (9.0-15.3)	11.0 (11.0-11.1)
Right No IMC	0	-	-	24	0.6 (0.6-1.1)	0.2 (0.2-0.3)	40	0.7 (0.7-0.7)	0.2 (0.2-0.2)	29	0.3 (0.3-0.3)	0.1 (0.1-0.1)
Right IMC	30	8.7 (8.7-8.9)	1.8 (1.8-1.9)	60	10.9 (7.9-14.3)	1.8 (1.0-1.9)	22	11.6 (9.1-12.9)	1.2 (0.9-1.5)	11	1.7 (1.7-9.4)	0.5 (0.5-1.1)
All women	72	8.9 (8.7-12.2)	2.8(1.8-8.9)	182	8.9 (4.8-16.4)	2.8 (1.6-7.7)	137	4.8 (0.7-10.5)	4.4 (0.3-7.2)	104	1.5 (0.3-4.8)	2.1 (0.1-2.2)

* The radiotherapy charts used for this study were from women included in case-contol studies of heart disease after breast cancer treatment. Similar dose changes per decade were observed when both cases and controls were included (see webtable 2).

Abbreviations: IMC: internal mammary chain, IQR: inter-quartile range

Table A.4. Whole heart and left ventricle radiation doses according to decade estimated for 276 individual women selected as cases for case-control studies of heart disease after treatment for breast cancer who underwent radiotherapy at the Netherlands Cancer Institute or the Erasmus MC Centre in the Netherlands during 1970-2009.*

	Treatment period											
	1970s			1980s			1990s			2000s		
Regions	No.	Median mean dose (IQR)		No.	Median mean dose (IQR)		No.	Median mean dose (IQR)		No.	Median mean dose (IQR)	
irradiated	women	Whole heart	Left ventricle	women	Whole heart	Left ventricle	women	Whole heart	Left ventricle	women	Whole heart	Left ventricle
Left No IMC	4	3.25 (3.25-3.5)	3.75 (3.75-4.2)	15	4.6 (4.3-4.8)	6.7 (6.5-7.2)	16	5.0 (4.7-4.8)	6.5 (6.5-7.2)	13	1.9 (1.5-1.6)	2.3 (2.1-2.2)
Left IMC	25	16.4 (12.0-22.5)	12.1 (8.7-17.6)	41	17.5 (8.3-26.5)	12.4 (7.1-20.0)	15	19.1 (17.2-21.0)	12.9 (10.4-15.3)	8	14.4 (14.5-16.1)	11.2 (11.3-11.4)
Right No IMC	3	1.9 (1.4-2.1)	0.7 (0.6-0.8)	14	0.7 (0.6-0.7)	0.2 (0.2-0.2)	9	1.4 (0.7-2.8)	0.3 (0.2-0.4)	18	0.3 (0.3-0.3)	0.1 (0.1-0.1)
Right IMC	28	10.1 (8.7-10.0)	1.7 (1.8-1.9)	41	11.5 (8.9-15.0)	1.6 (1.4-1.9)	16	13.1 (10.5-14.2)	1.4 (0.9-1.8)	10	7.8 (3.4-9.4)	1.0 (0.6-1.1)
All women	60	11.9 (8.7-13.2)	6.1 (1.8-8.9)	111	11.4 (4.8-16.5)	6.2 (1.6-8.9)	56	10.5 (4.8-16.8)	5.8 (1.2-7.7)	49	4.6 (0.3-9.0)	2.7 (0.1-2.2)

* The radiotherapy charts used for this study were from women included in case-contol studies of heart disease after breast cancer treatment. Similar dose changes per decade were observed when both cases and controls were included (see webtable 2).

Abbreviations: IMC: internal mammary chain, IQR: inter-quartile range

Table A.5. Mean radiation EQD2 doses to cardiac structures from typical breast cancer radiotherapy regimens used at the Netherlands Cancer Institute or the Erasmus MC Cancer Institute in the Netherlands during 1970-2009.

Radiotherapy regimen*						**Mean doses (Gy_2)†**											
						Whole heart		*Left ventricle*		*Pulmonary valve*		*Aortic valve*		*Mitral valve*		*Tricuspid valve*	
Decade of radiotherapy	*Medial border*	*Field arrangement*	*Usual Beam energies*	*Usual Dose Gy ‡*	*Dose per fraction*	*Left§*	*Right§*	*Left*	*Right*	*Left*	*Right*	*Left*	*Right*	*Left*	*Right*	*Left*	*Right*
Tangential fields (290 charts)																	
1970s	Midline	Tangents	250 keV¶	15.0	3	3	1	3	0	-	-	-		-	-	-	-
1970s-1980s	Midline	Tangents	Co^{60}	50.0	2.0	3.8	0.6	5.7	0.4	1.5	0.7	0.8	0.8	0.7	0.5	0.7	0.8
1980s-2000s	Midline	Tangents (Fig. 1a)	6 MV	50.0	2.0	3.8	0.4	5.8	0.1	1.1	0.3	0.6	0.5	0.5	0.2	0.5	0.6
2000s	1cm ipsi	Tangents (Fig. 1b)	6 MV	50.0	2.0	0.9	0.2	1.3	0.0	0.7	0.1	0.4	0.2	0.4	0.1	0.3	0.3
Megavoltage IMC fields (200 charts)																	
Megavoltage																	
1970s-1980s	1cm contra	Direct IMC	Co^{60}	43.0	2.9	19.3	12.5	13.9	1.0	34.4	2.4	30.0	25.5	23.1	2.0	26.7	31.2
1970s-1980s	1cm contra	Direct IMC, matching chest wall	Co^{60}/9 MeV	40.0/37.6	3.3/2.9	22.9	11.9	17.3	0.9	45.9	2.3	35.2	25.7	26.8	2.0	31.1	31.6
1970s-1980s	1cm contra	Direct IMC, matching tangents (Fig. 1c)	Co^{60}/6 MV	53.8/50.0	2.2/2.0	25.0	14.2	18.5	1.2	44.3	2.9	38.2	28.2	29.9	2.6	34.0	34.5
1970s-1990s	1cm contra	Direct IMC, direct SCF/axilla/lateral thorax	6 MV/ 8 MV	40.5/44.5	2.3/2.5	23.7	12.9	17.5	0.9	41.7	1.8	37.8	27.9	30.6	1.7	35.5	34.9
1980s-2000s	1cm contra	Direct IMC, matching tangents	6 MV/6 MV	50.0/50.0	2.0/2.0	31.1	17.3	22.9	1.0	53	1.7	48	34.4	38	1.8	44.8	45.4
Mixed megavoltage/electron																	
1980s	1cm contra	Direct IMC, matching tangents	Co^{60}/12 MeV/6 MV	24.0/22.0/46.0	2.0/2.0/2.0	11.8	8.1	6.8	0.1	28.7	2.4	17.7	14.6	11.1	1.3	13.3	14.5
1980s-1990s	1cm contra	Direct IMC, matching tangents	6 MV/12 MeV/6 MV	24.0/26.0/50.0	2.0/2.0/2.0	18.8	10.6	12.3	0.7	36	2.1	22.8	18.4	14.8	1.0	18.1	19.5
1990s-2000s	1cm contra	Direct IMC, matching chest wall (Fig. 1d)	6 MV/12 MeV/9 MeV	22.5/22.5/40.0	2.5/2.5/2.5	16.3	9.0	8.7	0.5	43	1.9	24.8	19.3	15.7	0.8	19.5	2.2
1990s-2000s	1cm contra	Direct IMC	6 MV/12 MeV	18.7/21.4	2.7/2.7	14.6	8.1	9.2	0.4	29	1.6	18	14.1	11	0.7	13.6	14.4
2000s	1cm contra	Direct IMC, matching tangents	6 MV/12 MeV/6 MV	18.0/32.0/50.0	2.0/2.0/2.0	12.0	6.9	7.5	0.6	27.7	2.4	14.6	13.5	8.0	1.0	10.1	11.7
2000s	2cm contra	Oblique IMC, matching tangents (Fig. 1e)	6 MV/12 MeV/6 MV	18.0/32.0/50.0	2.0/2.0/2.0	6.4	0.9	7.5	0.3	10	0.5	1.5	0.9	0.9	0.3	0.8	0.8
Anterior orthovoltage or mixed orthovoltage/megavoltage (172 charts)																	
1970s-1980s	1cm contra	Direct IMC (Fig 1f)	250 keV¶	37.2	2.5	12	9	6	1	-	-	-	-	-	-	-	-
1980s	1cm contra	Direct IMC	250 keV/Co^{60}||	17.2/22.7	2.5/2.8	-	-	-	-	-	-	-	-	-	-	-	-
1990s	1cm contra	Direct IMC	250 keV/6 MV||	23.5/24.0	1.8/2.0	-	-	-	-	-	-	-	-	-	-	-	-
Anterior electron (92 charts)																	
1970s-1980s	1cm contra	Direct IMC (Fig. 1g)	12 MeV	45.0	3.0	7.1	4.3	1.9	0.3	24.0	2.4	6.9	7.5	0.5	0.3	2.0	3.6
1970s-1980s	1cm contra	Direct IMC, matching tangents	12 MeV/6 MV	45.0/45.0	3.0/1.8	7.3	4.5	2.1	0.3	24.6	2.5	7.1	7.8	0.7	0.3	2.1	3.8
1970s-1990s	1cm contra	Direct chest wall	9 MeV	45.0	3.0	3.4	1.3	1.5	0.1	12.0	0.4	0.6	0.8	0.2	0.1	0.3	0.3
1970s-2000s	1cm contra	Direct chest wall, direct SCF/axilla/lateral thorax (Fig. 1h)	9 MeV/8 MV	46.0/50.0	2.0/2.2	4.5	1.8	2.7	0.2	13.6	0.6	2.1	2.2	1.4	0.3	1.5	1.5

Highlighted regimens are those used to irradiate the breast/chest wall and/or the SCF/axilla but not the IMC. All of the other regimens included the IMC.

*For further details on the radiotherapy regimens see webtable 1. Regimens a-h are illustrated in Fig. 1

†Mean cardiac doses estimated using manual planning are given to nearest Gy due to the uncertainties in dose estimation.

‡Usual total dose (100%) to the target regions (see webtable 1 for dose ranges). For direct regimens this was the Dmax. For tangents this was the dose delivered to the centre of the breast or chest wall apart from orthovoltage tangents where total dose was the skin dose at the surface of the breast.

§Cardiac doses from regimens used to irradiate left-breast and right-breast cancer.

¶ EQD2 doses were not estimated for the cardiac valves because these structures were too small to generate dose volume histograms from manual planning

|| No values are given for these mixed field techniques as the dose distribution from the orthovoltage field was generated using manual planning and the dose distribution from the megavoltage field was generated using CT-planning. It was not possible to generate combined dose-volume histograms from the two different planning methods.

Abbreviations: IMC: internal mammary chain, keV: kilovoltage, MV: megavoltage; MeV: mega electron-volts, SCF: supraclavicular fossa, contra: contralateral, ipsi: ipsilateral, Co^{60}: cobalt 60

Table A.6. Whole heart cardiac radiation doses from typical breast cancer radiotherapy regimens used at the Netherlands Cancer Institute of the Erasmus MC Cancer Institute in the Netherlands during 1970-2009.

Radiotherapy regimen*					Whole heart dose-volume measures (%) †															
Decade of radiotherapy	*Medial border*	*Field arrangement*	*Beam energy*	*Dose Gy‡*	V_{5Gy}		V_{10Gy}		V_{15Gy}		V_{20Gy}		V_{25Gy}		V_{30Gy}		V_{35Gy}		V_{40Gy}	
					Left §	*Right §*	*Left*	*Right*	*Left*	*Right*	*Left*	*Right*	*Left*	*Right*	*Left*	*Right*	*Left*	*Right*	*Left*	*Right*
Tangential fields (290 charts)																				
1970s	Midline	Tangents	250 keV	15.0	33	5	11	<0.1	<0.1	<0.1	<0.1	<0.1	<0.1	<0.1	<0.1	<0.1	<0.1	<0.1	<0.1	<0.1
1970s-1980s	Midline	Tangents	Co^{60}	50.0	16.8	<0.1	12.2	<0.1	10.1	<0.1	8.6	<0.1	7.4	<0.1	6.1	<0.1	4.6	<0.1	2.6	<0.1
1980s-2000s	Midline	Tangents (Fig. 1a)	6 MV	50.0	11.8	<0.1	9.1	<0.1	8.2	<0.1	7.6	<0.1	7.1	<0.1	6.6	<0.1	6.2	<0.1	5.4	<0.1
2000s	1cm ipsi	Tangents (Fig. 1b)	6 MV	50.0	3.0	<0.1	1.7	<0.1	1.3	<0.1	1.1	<0.1	1.0	<0.1	0.8	<0.1	0.6	<0.1	0.3	0.0
Megavoltage IMC fields (200 charts)																				
Megavoltage																				
1970s-1980s	1cm contra	Direct IMC	Co^{60}	43.0	80.8	46.4	74.1	41.5	69.5	38.5	64.0	35.6	45.5	31.3	22.0	20.8	3.6	6.9	<0.1	<0.1
1970s-1980s	1cm contra	Direct IMC,matching chest wall	Co^{60}/9 MeV	40.0/37.6	85.5	45.5	77.8	40.7	72.7	37.4	67.1	33.8	50.9	25.4	28.1	11.5	11.1	1.5	2.3	<0.1
1970s-1990s	1cm contra	Direct IMC, direct SCF, axilla, lateral thorax	6 MV/8 MV	40.5/44.5	85.3	42.9	73.3	40.0	69.6	38.5	67.8	36.9	66.0	35.8	58.3	34.2	34.1	29.4	11.5	17.6
1970s-1980s	1cm contra	Direct IMC, matching tangents (Fig. 1c)	Co^{60}/6 MV	53.8/ 50.0	87.6	48.5	78.3	43.4	73.8	40.5	70.4	38.0	66.7	35.8	60.8	32.9	44.8	27.1	27.9	17.2
1980s-2000s	1cm contra	Direct IMC ,matching tangents	6 MV/6 MV	50.0/50.0	82.8	42.0	72.8	38.2	70.8	37.5	69.2	36.6	67.7	35.8	66.2	35.1	64.5	34.0	54.4	31.0
Mixed megavoltage/electron																				
1980s	1cm contra	Direct IMC, matching tangents	Co^{60}/12 MeV/6 MV	24.0/22.0/46.0	79.7	45.0	60.1	39.1	29.0	32.7	21.1	19.4	15.6	12.9	10.9	8.4	6.5	4.5	2.3	1.4
1980s-1990s	1cm contra	Direct IMC, matching tangents	6 MV/12 MeV/6 MV	24.0/26.0/50.0	80.0	41.8	71.8	38.0	68.2	36.1	57.0	31.7	30.2	19.6	21.8	13.8	16.2	9.9	11.5	6.5
1990s-2000s	1cm contra	Direct IMC, matching chest wall (Fig. 1d)	6 MV/12 MeV/9 MeV	22.5/22.5/40.0	65.9	34.7	58.3	30.3	53.4	28.0	39.4	57.0	23.0	13.6	16.3	9.7	11.2	6.8	7.3	4.1
1990s-2000s	1cm contra	Direct IMC	6 MV/12 MeV	18.7/21.4	73.9	35.4	68.9	30.8	59.0	28.3	27.6	24.8	19.1	15.5	12.9	11.3	7.0	8.4	1.6	5.9
2000s	1cm contra	Direct IMC, matching tangents	6 MV/12 MeV/6 MV	18.0/32.0/50.0	81.4	43.7	73.0	39.1	36.7	23.3	26.0	16.2	20.1	12.0	15.1	8.5	10.5	5.5	6.1	2.7
2000s	2cm contra	Oblique IMC, matching tangents (Fig. 1e)	6 MV/12 MeV/6 MV	18.0/32.0/50.0	44.8	7.5	38.5	2.6	22.9	1.0	13.8	0.4	8.9	0.1	5.2	0.0	2.4	0.0.	1.2	0.0
Orthovoltage or mixed orthovoltage/megavoltage IMC fields (172 charts)																				
1970s-1980s	1cm contra	Direct IMC (Fig. 1f)	250 keV	37.2	99	89	78	59	61	38	38	23	18	10	8	6	2	2	0	0
1980s	1cm contra	Direct IMC ¶	250 keV/Co^{60}	17.2/22.7	-	-	-	-	-	-	-	-	-	-	-	-	-	-	-	-
1990s	1cm contra	Direct IMC ¶	250 keV/6 MV	23.5/24.0	-	-	-	-	-	-	-	-	-	-	-	-	-	-	-	-
Electron chest wall or IMC fields (92 charts)																				
1970s-1980s	1cm contra	Direct IMC (Fig. 1g)	12 MeV	45.0	35.0	23.2	26.1	16.7	20.3	12.6	15.9	9.4	12.0	6.8	8.4	4.4	5.0	2.3	1.7	0.4
1970s-1980s	1cm contra	Direct IMC, matching tangents	12 MeV/6 MV	45.0/45.0	36.7	23.6	26.3	16.9	20.2	12.8	15.6	9.6	11.8	6.9	8.4	4.6	5.1	2.4	2.1	0.5
1970s-1990s	1cm contra	Direct chest wall	9 MeV	45.0	19.3	9.2	13.9	5.8	10.4	3.6	7.7	2.2	5.5	1.1	3.7	0.3	2.2	0.0	1.0	0.0
1970s-2000s	1cm contra	Direct chest wall, direct SCF, axilla, lateral thorax (Fig.	9 MeV/8 MV	46.0/50.0	24.8	11.2	16.5	7.0	12.3	4.7	9.2	3.2	6.8	2.0	4.8	1.1	3.2	0.5	1.8	0.1

Highlighted regimens are those used to irradiate the breast/chest wall and/or the SCF/axilla but not the IMC. All of the other regimens included irradiation to the IMC.
*For further details on the radiotherapy regimens see webtable 1. Regimens a-h are illustrated in Fig. 1
† V_{XGy} (%): the percent volume of the whole heart receiving X Gy.
‡Usual total dose (100%) to the target regions (see webtable 1 for dose ranges). For direct regimens this was the Dmax. For tangential regimens this was the dose delivered to the centre of the breast or chest wall apart from orthovoltage tangents where the total dose was the skin dose at the surface of the breast.
§Cardiac doses from regimens used to irradiate left-sided and right-sided breast cancer.
¶ No values are given for these mixed field techniques as the dose distribution from the orthovoltage field was generated using manual planning and the dose distribution from the megavoltage field was generated using CT-planning. It was not possible to generate combined dose-volume histograms from the two different planning methods.
Abbreviations: keV: kilovoltage, MV: megavoltage; MeV: mega electron-volts, lat: lateral, SCF: supraclavicular fossa, IMC: internal mammary chain, contra: contralateral, ipsi: ipsilateral, Co^{60}: cobalt 60

Table A.7. Comparison of doses in this study with previously published estimates for comparable regimens used in the Netherlands during 1970-2009.

Radiotherapy regimen*					Whole heart doses							
					Left-sided regimens						*Right-sided regimens*	
Years of radiotherapy	*Medial border*	*Field arrangement*		*Usual Dose Gy †*	*Mean (Gy)*		*V_{10Gy} (%)*		*V_{40Gy} (%)*		*Mean (Gy)*	
					Duane	*Van der Laan*	*Duane*	*Hurkmans*	*Duane*	*Hurkmans*	*Duane*	*Van der Laan*
Tangential fields (290 charts)												
1970s	Midline	Tangents	250 keV	15.0	-	-	-	-	-	-	-	-
1970s-1980s	Midline	Tangents	Co^{60}	50.0	-	-	-	-	-	-	-	-
1980s-2000s	Midline	Tangents (Fig. 1a)	6 MV	50.0	-	-	-	-	-	-	-	-
2000s	1cm ipsi	Tangents (Fig. 1b)	6 MV	50.0	-	-	-	-	-	-	-	-
Megavoltage IMC fields (200 charts)												
Megavoltage												
1970s-1980s	1cm contra	Direct IMC	Co^{60}	43.0	-	-	-	-	-	-	-	-
1970s-1980s	1cm contra	Direct IMC, matching chest wall	Co^{60}/9 MeV	40.0/37.6	-	-	-	-	-	-	-	-
1970s-1980s	1cm contra	Direct IMC, matching tangents (Fig. 1c)	Co^{60}/6 MV	53.8/50.0	-	-	-	-	-	-	-	-
1970s-1990s	1cm contra	Direct IMC, direct SCF/axilla/lateral thorax	6 MV/8 MV	40.5/44.5	-	-	-	-	-	-	-	-
1980s-2000s	1cm contra	Direct IMC, matching tangents	6 MV/6 MV	50.0/50.0	-	-						
Mixed megavoltage/electron												
1980s	1cm contra	Direct IMC, matching tangents	Co^{60}/12 MeV/6 MV	24.0/22.0/46.0	-	-	-	-	-	-	-	-
1980s-1990s	1cm contra	Direct IMC, matching tangents	6 MV/12 MeV/6 MV	24.0/26.0/50.0	-	-	-	-	-	-	-	-
1990s	1cm contra	Direct IMC, matching chest wall§ (Fig 1d)	6 MV/12 MeV/9 MeV	22.5/22.5/40.0	-	-	-	-	-	-	-	-
1990s-2000s	1cm contra	Direct IMC	6 MV/12 MeV	18.7/21.4	-	-	-	-	-	-	-	-
2000s	1cm contra	Direct IMC, matching tangents	6 MV/12 MeV/6 MV	18.0/32.0/50.0	16.1	12.9	73	60	6	4	9.4	7.2
2000s	2cm contra	Oblique IMC, matching tangents (Fig. 1e)	6 MV/12 MeV/6 MV	18.0/32.0/50.0	-	-	39	30	1	2	-	-
Orthovoltage or mixed orthovoltage/megavoltage IMC fields (172 charts)												
1970s-1980s	1cm contra	Direct IMC (Fig. 1f)	250 keV	37.2	-	-	-	-	-	-	-	-
1980s	1cm contra	Direct IMC	250 keV/Co^{60}	17.2/22.7	-	-	-	-	-	-	-	-
1990s	1cm contra	Direct IMC	250 keV/6 MV	23.5/24.0	-	-	-	-	-	-	-	-
Electron chest wall or IMC fields (92 charts)												
1970s-1980s	1cm contra	Direct IMC (Fig. 1g)	12 MeV	45.0	-	-	-	-	-	-	-	-
1970s-1980s	1cm contra	Direct IMC, matching tangents	12 MeV/6 MV	45.0/45.0	8.3	7.5	-	-	-	-	5.3	4.3
1970s-1990s	1cm contra	Direct chest wall	9 MeV	45.0	-	-	-	-	-	-	-	-
1970s-2000s	1cm contra	Direct chest wall, direct SCF/axilla/lateral thorax (Fig.	9 MeV/8 MV	46.0/50.0	-	-	-	-	-	-	-	-

V10Gy (%): the percent volume of the whole heart receiving 10 Gy.

V40Gy (%): the percent volume of the whole heart receiving 40 Gy.

*For further details on the radiotherapy regimens see Webtable 1. Regimens a-h are illustrated in Fig. 1

†Usual total dose (100%) to the target regions (see Webtable 1 for dose range). For direct regimens this was the Dmax. For tangential regimens the total dose (100%) was delivered to the centre of the breast or chest wall apart from orthovoltage tangents where the total dose was the skin dose at the surface of the breast.

Abbreviations: IMC: internal mammary chain keV: kilovoltage, MV: megavoltage; MeV: mega electron-volts, SCF: supraclavicular fossa, contra: contralateral, ipsi: ipsilateral, Co60: cobalt 60

Hurkmans CW, Saarnak AE, Pieters BR, Borger JH, Bruinvis IA. An improved technique for breast cancer irradiation including the locoregional lymph nodes. Int J Radiat Oncol Biol Phys 2000;47:1421-9.

van der Laan HP, Dolsma WV, van 't Veld AA, et al. Comparison of normal tissue dose with three-dimensional conformal techniques for breast cancer irradiation including the internal mammary nodes. Int J Radiat Oncol Biol Phys 2005;63:1522-1530

Table A.8. Comparison of doses per decade in this study with previously published doses per decade in the Netherlands during 1970-1986.

	Mean heart dose (Gy) (range)	
Treatment period	*Hooning**	*Duane†*
**1970-1979**		
RT fields		
Chest/breast: right-sided	~3 (1.2-3.8)	~2 (1.4-2.8)
Chest/breast: left-sided	~7 (2.5-9.0)	~3 (2.1-4.2)
IMC only: right-sided	7 (0.5-11.6)	~9 (5.1-19.8)
IMC only: left-sided	~9 (0.7-15.6)	~14 (7.9-35.2)
IMC + chest wall/breast: right-sided	~11 (2.7-15.4)	~10 (4.7-18.5)
IMC + chest wall/breast: left-sided	~15 (4.7-18.3)	~13 (3.5-28.8)
**1980-1986**		
RT fields		
Chest/breast: right-sided	~1.5 (1.2-1.6)	~1 (0.6-2.8)
Chest/breast: left-sided	~5 (2.5-5.3)	~5 (4.0-6.3)
IMC only: right-sided	~6 (0.5-11.6)	~9 (1.0-14.6)
IMC only: left-sided	~7 (0.7-15.6)	~15 (7.9-35.2)
IMC + chest wall/breast: right-sided	~9 (2.5-14.0)	~13 (1.3-16.6)
IMC + chest wall/breast: left-sided	~13 (4.0-19.9)	~19 (4.0-37.5)

*Doses were estimated by reconstructing typical field borders, beam energies and total dose for 14 commonly used regimens on a typical patient. These doses were weighted according to frequency c use in the 1970-1979 and 1980-1986.[1]

†Heart doses were estimated by reconstructing 44 regimens based on information from the radiothe charts of 771 women. Dose volume histograms were produced for each individual woman taking into account the total dose each woman received.

Abbreviations: IMC: internal mammary chain

[1] Hooning MJ, Botma A, Aleman BM,,et al. Long-term risk of cardiovascular disease in 10-year survivc of breast cancer. Journal of the National Cancer Institute. 2007; 99:365-75.
